# Application of AI for Short-Term PV Generation Forecast

**DOI:** 10.3390/s24010085

**Published:** 2023-12-23

**Authors:** Helder R. O. Rocha, Rodrigo Fiorotti, Jussara F. Fardin, Hilel Garcia-Pereira, Yann E. Bouvier, Alba Rodríguez-Lorente, Imene Yahyaoui

**Affiliations:** 1 Department of Electrical Engineering, Federal University of Espírito Santo, Av. Fernando Ferrari, 514, Vitória 29075-910, ES, Brazil; helder.rocha@ufes.br (H.R.O.R.); rodrigo.fiorotti@ifes.edu.br (R.F.); jussara.fardin@ufes.br (J.F.F.); 2 Department of Electrical Engineering, Federal Institute of Espírito Santo, São Mateus 29932-540, ES, Brazil; 3 Higher School of Experimental Sciences and Technology, University of Rey Juan Carlos, 28933 Madrid, Spain; hilel.garcia@urjc.es (H.G.-P.); yann.bouvier@urjc.es (Y.E.B.); alba.rodriguez@urjc.es (A.R.-L.)

**Keywords:** photovoltaic power, short-term forecast, artificial intelligence, LSTM, BILSTM, TCN

## Abstract

The efficient use of the photovoltaic power requires a good estimation of the PV generation. That is why the use of good techniques for forecast is necessary. In this research paper, Long Short-Term Memory, Bidirectional Long Short-Term Memory and the Temporal convolutional network are studied in depth to forecast the photovoltaic power, voltage and efficiency of a 1320 Wp amorphous plant installed in the Technology Support Centre in the University Rey Juan Carlos, Madrid (Spain). The accuracy of these techniques are compared using experimental data along one year, applying 1 timestep or 15 min and 96 step times or 24 h, showing that TCN exhibits outstanding performance, compared with the two other techniques. For instance, it presents better results in all forecast variables and both forecast horizons, achieving an overall Mean Squared Error (MSE) of 0.0024 for 15 min forecasts and 0.0058 for 24 h forecasts. In addition, the sensitivity analyses for the TCN technique is performed and shows that the accuracy is reduced as the forecast horizon increases and that the 6 months of dataset is sufficient to obtain an adequate result with an MSE value of 0.0080 and a coefficient of determination of 0.90 in the worst scenarios (24 h of forecast).

## 1. Introduction

The demographic growth is principally the main cause for the increase in the required electrical power. This means the need for using new energy sources and techniques that optimize their use [1,2]. Moreover, Distributed Generation (DG), which consists of generating energy near to the place of consumption, allows losses to be reduced since the power transport stage is avoided and therefore, additional energy costs are avoided [3]. Thus, many research papers focuses on using PV panels, such as amorphous panels in a DG application. In fact, the interest of using them has been rising especially in residential applications since they are characterized by their flexibility and the ease in installation even in complicated surfaces like in roofs or non-flat surfaces. In addition, they are more economic compared with monocrystalline and polycrystalline PV panels [4]. Furthermore, amorphous PV panels have the possibility to generate energy with low radiation levels, which makes them suitable for low radiation regions or shaded zones. Despite the already detailed advantages, amorphous panels are characterized by less efficiency, compared to monocrystalline and polycrystalline PV panels. Therefore, the selection of the PV panels’ technology should be based on the available surface of the plant, the economic constraints and the total PV power to be installed. Photovoltaic (PV) energy is characterized by the intermittence of the power generation in comparison with others traditional power generation technologies. To deal with it, a possible solution that can be applied consists in forecasting the power to be generated by the PV plant [5]. Indeed, a good forecast of the PV power allow the electrical loads supplied to be known, an efficient use of the produced energy, an optimized cost of the plant and a good operation of the grid to be obtained [6]. In this sense, many methods are used and that are broadly categorized in physical and statistical models. In fact, the first ones depend on the solar radiation and the ambient temperature, whereas numerical weather forecast (NWP) [7] leans on environmental measurements. Indeed, in [8], the forecast precision is improved by including the effects of the weather characteristics, such as wind direction, strength and temperature. This reduces the dependence on a single factor. Moreover, cloud coverage estimation is also used in [9] to reduce this effect on the solar irradiation. These improvements, however, increase the required computing power and the correlation between the different types of weather data can still be unclear. On the other hand, the statistical techniques are very diverse and also used for forecast, namely ARIMA [10,11], Bayesian statistics [12,13] and Markov chains [14,15].

Modern methods that include Artificial Neural Networks (ANNs) and deep learning models are widely used for PV power forecasting. The adaptability and capacity to model temporal dependencies make the LSTM a relevant ANN technique for many applications, namely in healthcare applications like optimizing treatment plans or better recognition of tumors [16,17]. Also, it is used in applications related with electric vehicles for the trajectory forecast [18]. The LSTM have also demonstrated their usefulness in various applications within the energy sector, especially for tasks related to electrical grid optimization, as they help in facilitating the development of strategies for capacity planning and operational management integrating forecasting and even fault detection [19,20]. In this scenario, LSTM have proven to outperform other architectures as it is suitable to forecast intermittent variables that are time depending, like the case of PV power [21,22,23].

The Bidirectional Long Short-Term Memory (BILSTM) is an evolution of the LSTM where the temporal structure considers the bidirectional relationship of the input data. In general, BiLSTM is more accurate than one-way LSTM [24]. Indeed, this algorithm is very flexible and used in several applications namely in text analysis, fault identification [25], etc. This technique fulfilled some success in the field of PV energy forecast [26,27,28].

Also, convolutional architecture designed for sequential modeling called Temporal Convolutional Network (TCN) is also used for forecast tasks like in [29], where higher accuracy is obtained. A related literature review shows the application of TCN for electricity load [30] and electricity price [31] forecasting. For wind energy applications, TCN is applied to forecast wind power which depends on the wind speed data [32,33]. Moreover, deep learning strategies (that include TCN) are also used for solar power forecasting [34,35] by the forecast of the solar radiance. Following this trend on PV generation, Ref. [36] compares different deep learning methods for PV generation (TCN included). Ref. [37] focus on a hybrid architecture that includes TCN for very short-term forecasting.

Moreover, to evaluate the prediction results in different horizons, the LSTM and Grid Search Algorithm (GSA-LSTM) methods have been applied together in [38] to forecast the PV power output, varying from 1 h to 2 months. The results show that the accuracy tends to decrease as the forecasting period increases, i.e, the longer the forecast horizon, the more difficult high accuracy forecast is. Given these promising deep learning techniques, this research paper aims to apply:

- LSTM, BILSTM and TCN to forecast, with the expanded windows of 96 samples, the power, efficiency and voltage of an amorphous PV plant;
- for a 15 min and subsequent 24 h time horizon;
- the same architecture per type of neural network is used to estimate the subsequent 15 min and 24 h time horizons using only the historical solar radiance and ambient temperature data as inputs.

The case study data used in this work are obtained from the amorphous PV plant installed at the Technical Support Centre of the University Rey Juan Carlos (Madrid, Spain). Then, the ANN techniques are applied and the obtained results are compared to study their effectiveness and accuracy.

The article is organized as follows: the state of the art is described in Section 3. Then, the application of LSTM, BILSTM and TCN is studied in depth in Section 3.3, whereas the results and discussion are provided in Section 4. Then, the paper ends by the conclusions section and the future works that are detailed in Section 5.

## 2. Related Works

For short-term forecasting, the estimated range varies from a few minutes to 24 h. The objective of this section is to study three ANN techniques to forecast the power, efficiency and voltage of the photovoltaic plant in period of T+1/Day+1, based on the parameters from period T/Day of solar radiation and ambient temperature. Therefore, LSTM, BILSTM and TCN have been selected as time-series forecasting algorithms, whose performances have been compared using the Mean Squared Error (MSE). The determination coefficient ($R^{2}$) is also applied to decide about the most suitable ANN technique between the aforementioned techniques to forecast the PV plant generation.

### 2.1. Long Short-Term Memory (LSTM)

LSTM networks are neural network models widely used for time-series forecast applications. Moreover, they are able to form a deeper network to enhance the learning step [39]. Since it a specialized form of a recurrent neural network (RNN), LSTM can learn thousands of timesteps compared to the previous 5–10 timesteps. This is achieved by incorporating a memory block or state cell that allows new data to be selectively stored or forgotten and information to be preserved without corrupting it [21].

**Figure 1:**
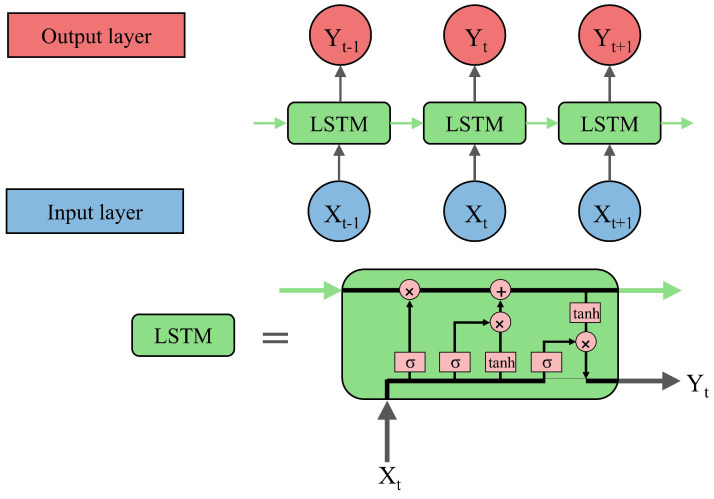
Principle of Long Short-Term Memory (LSTM).

In fact, it is composed of gates characterized by their ability to substitute not important data by more relevant new ones [40]. The operation of the LSTM requires the use of three blocs of data which are input vectors, the network’s last response and stored network memory. Indeed, the input vector and the last response of the network are necessary to describe the gate operation, while the forget gate is where the decision of eliminated or saved memory is taken. Then, the input gate adds updated data. Finally, the output gate is where decisions of the LSTM network outputs are taken. This operation of LSTM can be repeated to enhance the forecast performance. The structure of a basic architecture and an LSTM cell is depicted in Figure 1.

### 2.2. Bidirectional Long Short-Term Memory (BILSTM)

**Figure 2:**
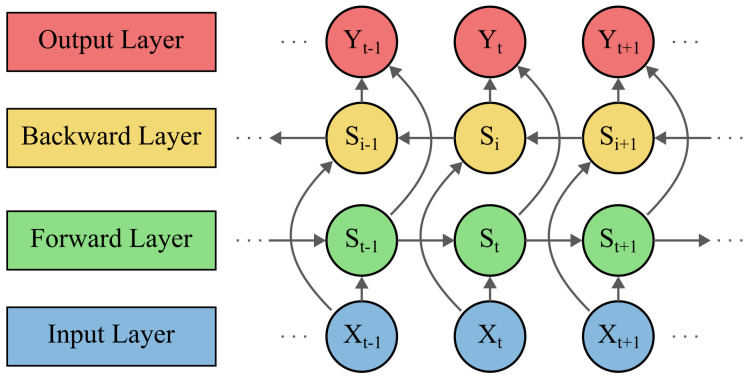
Principle of Bidirectional Long Short-Term Memory (BILSTM).

The learning capability of the LSTM model is enhanced by the introduction of the BILSTM, where the temporal structure considers the bidirectional relationship of the input data. In fact, it is composed of two LSTMs. The first one obtains the input in the forward direction. However, the second one obtains it in the backward direction [41]. The basic architecture of BILSTM is shown in Figure 2.

The regular LSTM method solves the problem of disappearing gradients. For an enhanced performance, the BiLSTM adds a bidirectional flow of information, moving in the forward and backward directions as depicted in Figure 2. The regular LSTM method improves the capability to store past data. Since the BILSTM consists of an LSTM in both directions of the information flow, the forecast accuracy is enhanced by considering both historical information (past) and trend information (future). In [42,43], the BiLSTM method is compared to others and it shows a higher accuracy by having the lowest errors RMSE, MAE and MAPE, making the BiLSTM an effective and reliable method for the PV generation forecast.

### 2.3. Temporal Convolutional Network (TCN)

**Figure 3:**
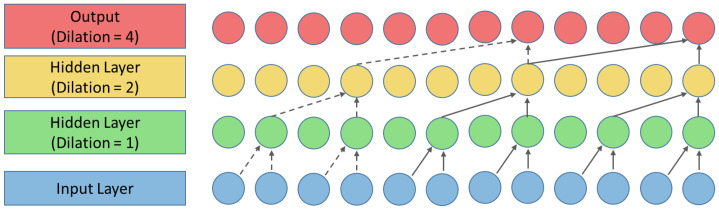
Principle of Temporal Convolutional Networks (TCN) with dilations [1, 2, 4].

Among convolutional networks’ architectures for the time-series forecast, TCNs stand out as typically achieving the best performance. The main characteristic of TCN is that it utilizes one-dimensional (1D) convolutional layers with dilated convolutions, allowing TCN to successfully identify possible short- and long-term reliance on the input data. According to [44], some of the advantages of using TCNs for sequence modeling includes that convolutions can be performed in parallel, it is a flexible architecture, uses stable gradients, requires reduced memory for operation and can take data with variable lengths in a recurrent way. Figure 3 shows a TCN architecture with dilations [1, 2, 4].

These characteristics make TCN appropriate for a wide range of applications that involve sequential data analysis. Some examples include human actions detection and actions segmentation [45], speech recognition [46], sentence embedding to process language [47], categorizing videos [48], medical purposes such as skeleton-based recognition [49], modern traffic flow forecasts [50] and even weather forecasting [51].

Thanks to how useful TCNs are for forecasting purposes, renewable energy-related forecasting is a very interesting application of TCN since the forecasts’ accuracy is important for economic reasons to ensure the electrical supply and to safely integrate and control an increasing number of renewable energy generation in electrical grids. As such, TCNs show that they can be an invaluable tool to process and forecast variables related to renewable energy and particularly solar power generation, where forecasting is useful both in the short term and long term.

## 3. Application of LSTM, BILSTM and TCN in the Amorphous Photovoltaic System Panels

### 3.1. Presentation of the PV Plant Characteristics

Amorphous silicon cells have the advantage of being more flexible and lighter, allowing a greater versatility when applied in different types of surfaces, including curved and flexible ones. This makes them an attractive option for integration into various devices and structures, such as smart clothing, buildings and other renewable energy devices. However, faster degradation compared to crystalline silicon cells is a significant concern and requires further research to improve the durability and lifespan of these cells.

**Figure 4:**
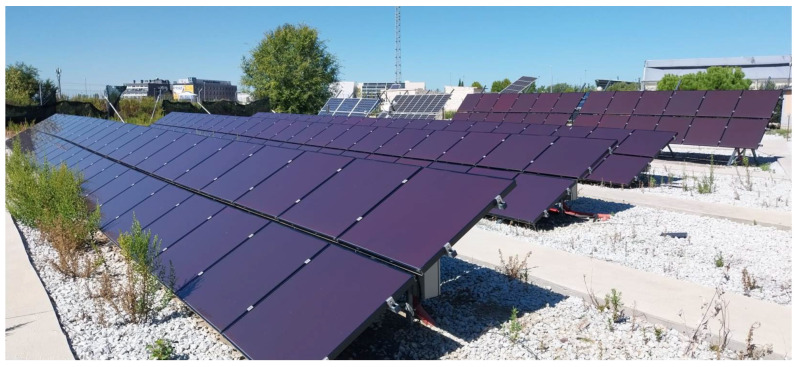
Amorphous System Photovoltaic Experimental Setup.

Figure 4 describes the 1320 Wp of installed amorphous photovoltaic plant which is the case of study of this research paper. The historical data was obtained by the monitoring system for a period between 1 September 2021, 00:00 h and 5 August 2022, 14:00 h, corresponding to 32,501 input patterns containing current, voltage, ambient temperature and irradiance sensors measured using the inverter.

Hence, the technical specifications of the amorphous photovoltaic module used in this study, the Kaneka G-EA060 sourced by Technosun (Paterna, Spain) is described in Table 1.

**Figure 5:**
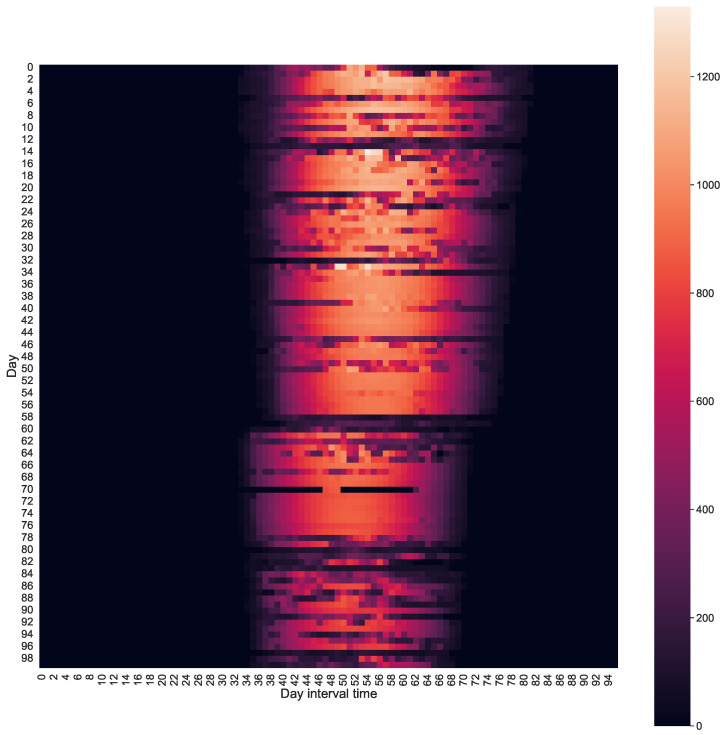
Amorphous System Photovoltaic Power Heatmap Over 100 Days.

Figure 5 presents the heat map of power production from the photovoltaic panel over 100 days compared to one day in a 15 min period. It can be seen that from day 58, the heat map shifted to the left due to the entry into force of winter time in Spain. It can also be noted that the darker lines are days with a lot of cloudiness, which leads to low energy production.

### 3.2. Dataset Preprocessing

The dataset used in this research paper are measured every 15 min and is obtained from the amorphous photovoltaic plant (previously described). The available measurement period was collected between 1 September 2021, 00:00 h and 5 August 2022, 14:00 h, corresponding to 32,501 input patterns containing the following variables: power (W), efficiency (kWh/kWp), voltage (V), irradiance (W/m${}^{2}$) and temperature (°C). The statistical analysis of dataset are shown in Table 2.

As it can be seen in Table 2, the temperature value reaches a maximum of 65 °C because the measurements are taken at the location where the inverter is installed, which has high heat dissipation and causes rise of the temperature. Since these measurements doe not correspond to the ambient temperature value at the location where the modules are installed and the TCN is designed to work primarily with just one input variable, therefore it has been removed from the dataset.

**Figure 6:**
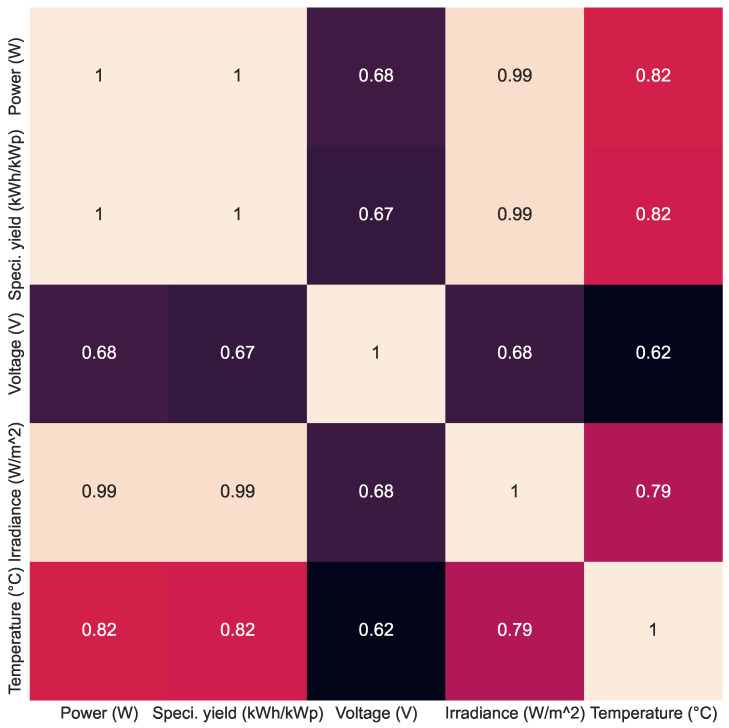
Correlation matrix of the variables’ power, efficiency, voltage, irradiance and temperature.

Figure 6 presents the heatmap of the variables’ power, efficiency, voltage, irradiance and temperature sensors that compose the dataset. It is observed that PV power and efficiency have a maximum relationship, where power strongly depends on irradiance. However, the correlation with temperature is 0.82. Voltage has an average correlation with all other variables in the dataset.

Before training, validating and testing ANN models, the data must be preprocessed to remove possible outliers and missing data. To remove outliers, the movemean method was used and some bounds were applied to the variables (e.g., irradiance < 0). When the outliers are identified, they are replaced by a Not a Number (NaN) to be read as missing data. To fill in outliers and missing data, the shape-preserving piecewise cubic spline interpolation was applied. In this dataset, there were only four missing data intervals and one outlier value was found in the irradiance variable, i.e., the data did not require any complex preprocessing.

### 3.3. Network Architectures, Hyperparameters and Train Process of Forecast Models

After conducting a detailed recurrent neural networks’ (RNNs) calibration experiment, the final architectures are presented in Table 3, Table 4 and Table 5. In Table 3, following two LSTM layers, there are two Dense layers, succeeded by a dropout layer with a value of 0.1. In Table 4, subsequent to the BiLSTM layer, there are two Dense layers, followed by a dropout layer with a value of 0.05. In Table 5, the TCN layer is composed of 32 filters, a kernel size of 6, ReLu activation and dilations of 1, 2, 4, 8, 16 and 32, respectively, with 1 NbStacks.

The evaluations have been performed using Python 3.12.1, Keras 3, and an Intel^®^ Core™ i7-12700H computer with a 2.2 GHz CPU and 16 GB of RAM (Intel, Santa Clara, CA, USA). For the execution of the computational experiments with the Recurrent Neural Networks (RNNs), meteorological data measured every 15 min were obtained between 1 September 2021, 00:00 h and 5 August 2022, 14:00 h, corresponding to 32,501 input patterns. Each input pattern of the RNNs contains the following variables: date, time and solar radiance (W/m${}^{2}$).

The photovoltaic system has a nominal power of 1320 Wp and contains power and voltage sensors integrated into the inverter, which will serve as the output variables for the RNNs, in addition to the efficiency variable (calculated indirectly). Subsequently, these variables were shifted by 15 min or 24 h (depending on the forecast horizon to perform), forming a complete pattern containing irradiance data at instant T=15 min (network input) with the power, voltage and efficiency at instant T+1 (desired network output) and day D=hour *k* (network input) with the power, voltage and efficiency at day D+1 h *k* (desired network output) for forecasting one day ahead.

The input and output data have been normalized into the range between 0 and 1 (min–max normalization) to help the CNNs to enhance their performance. Subsequently, the data has been divided into three sets: training (80%), validation (10%) and testing (10%). Finally, the test set has been used to measure the quality of the forecasts generated by the neural networks, using the following metrics for result analysis: MSE and $R^{2}$.

In addition, the training has been conducted using the Adam optimizer from the Keras library, with a batch size of 32 examples for 50 epochs. A technique for reducing the learning rate is used. It started at 0.002 and decreased during training, with a patience of 4, a factor of 0.6 and a minimum learning rate of 0.0001, while the hyperparameters are obtained after a minucious succession of empirical tests. The mean squared error (MSE) has been applied as the loss function, which is typically employed in regression problems. Finally, the Model Checkpoint function was utilized to save the weights, allowing them to be used later for making forecasts on new data.

**Figure 7:**
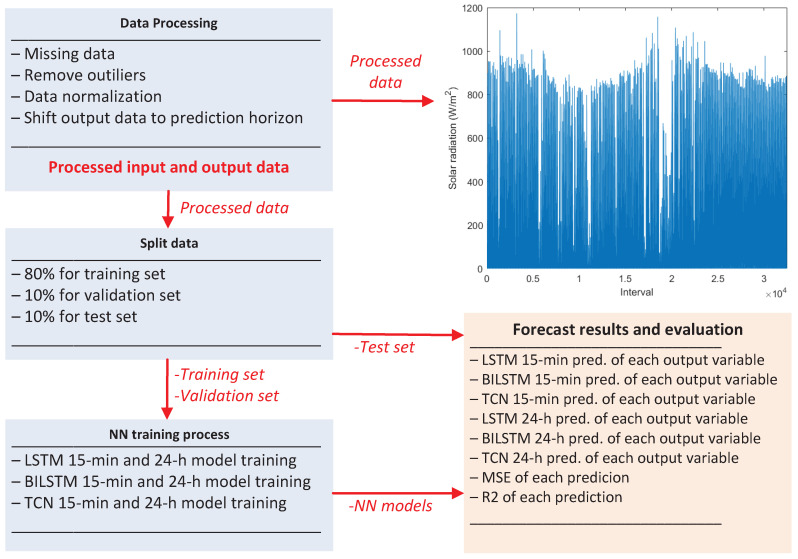
Overview of the developed framework.

After presenting and modeling each step of LSTM, BILSTM and TCN to forecast the power, efficiency and voltage of an amorphous PV system for a 15 min and 24 h time horizon, an overview of the developed framework is shown in Figure 7.

## 4. Results and Discussion

The results of the forecast simulations for a 15 min period and the subsequent 24 h using the RNNs are presented in Table 6 and Table 7. Both tables display the MSE, $R^{2}$ scores and execution times of the three forecasting models. The results are compared when replicating the data from period *T* or Day in period T+1 or Day+1. The table shows that the TCN neural network achieved better results in both forecasts.

Comparing the simulations of the 15 min horizon forecast in Table 6, it appears that all proposed neural networks obtained considerably better results than method *T* in T+1 as they obtained MSE results lower than 0.0053 in the test set, that is, the application of these artificial intelligence techniques managed to improve the performance using a rule that is simple to forecast. It is also worth noting that TCN is the network with the most satisfactory results, with an MSE value of 0.0024, which is significantly lower than the values obtained from LSTM and BILSTM. Regarding the computational efficiency of the models during the testing phase, all models are executed very quickly, ranging from 6 to 10 ms, making this not a problem when implementing any of the models in real applications.

Observing the values of Table 7, in the results of the 15 min horizon forecast, it has the same conclusions, i.e., the TCN is the network with the most satisfactory results, with an MSE value of 0.0024 and the LSTM and BILSTM have better values compared than the simple method Day in Day+1. As TCN obtained the best forecast results, its results will be presented separately and broken down for each of the target variables (power, efficiency and voltage) for both forecast horizons, as shown in Table 8.

Observing the results of Table 8, it appears that the forecast on the 15 min horizon gives better results than the 24 h horizon for all the variables, a fact that was expected since the temporal dependence between the latest input data (irradiation) and the variables to be predicted in the 15 min horizon is much greater than in 24 h, making it easier to obtain good results. In addition, the MSE values of power and efficiency are almost the same (because their correlation is 1) and higher than the voltage variable in both horizons; a fact that explains this result is the fact that the voltage has lower values of correlation with the input variables (as can be seen in Figure 6).

### 4.1. TCN Forecast Results in the 15 Min Horizon

**Figure 8:**
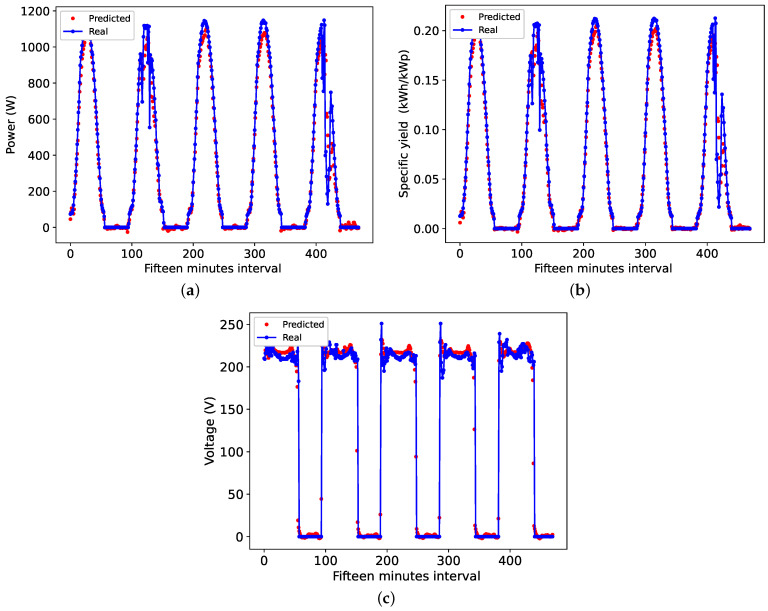
Forecast curve in 15 min: (**a**) Power, (**b**) efficiency and (**c**) Voltage.

To display the individual results of the variable forecast on the 15 min horizon, Figure 8a–c present a comparison between the actual data and the TCN-predicted data. These figures show a slight discrepancy between the curves obtained due to the presence of clouds over the system, as such moments of intermittency in photovoltaic generation are usually the most difficult to predict. It is observed that on the second and fifth days of forecast, there is a sharp variation in the estimated variables caused by the presence of cloudiness, which increases the difficulty of the forecast.

**Figure 9:**
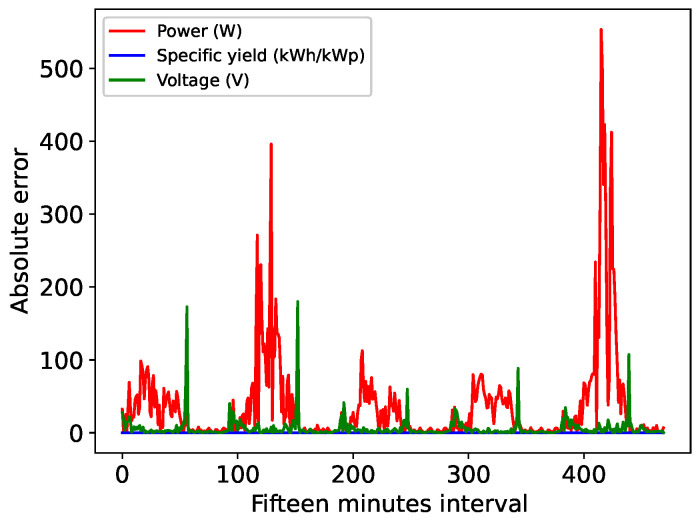
Absolute error for 15-min TCN forecast.

Figure 9 illustrates the absolute error in the TCN’s 15 min horizon forecasts for power, efficiency and voltage, revealing an increased forecast error during cloudy days, particularly for the power variable. As mentioned earlier, the second and fifth forecast days exhibit significant fluctuations in the estimated variables due to cloud cover, intensifying the complexity of the forecast process, as demonstrated in the absolute error depicted in Figure 9. Notably, on the fifth day, there was a point where the absolute error surpassed 500 W. If the presence of cloud cover persists for periods longer than 15 min, the TCN can rectify the forecast error.

**Figure 10:**
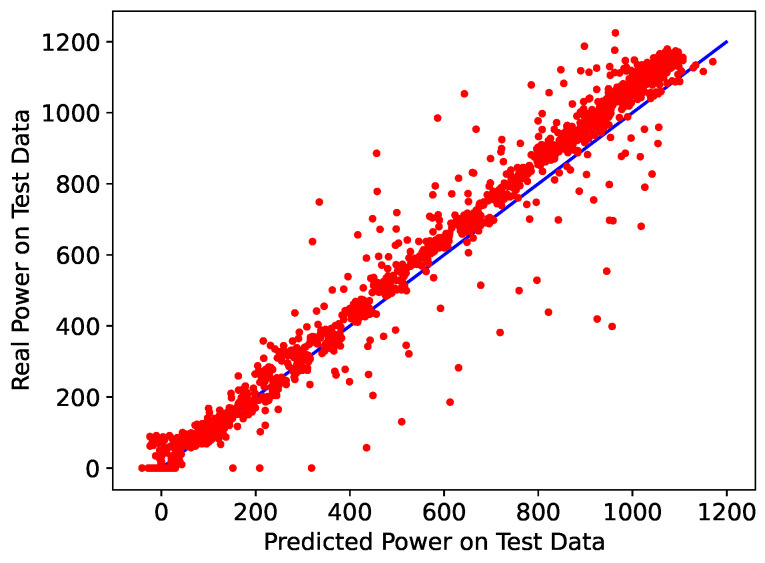
Real versus Predict Power ($R^{2}$) for 15 min using TCN forecast.

Figure 10 shows the simple linear regression between the predicted values (X-axis) and the actual values (Y-axis) for the TCN. From the regression, one can observe the fit of the obtained results, revealed by the alignment of the points and the line (red and blue). These are signs that the choice generates a high-quality forecasting model in which errors are minimized.

### 4.2. TCN Forecast Results in the 24 h Horizon

**Figure 11:**
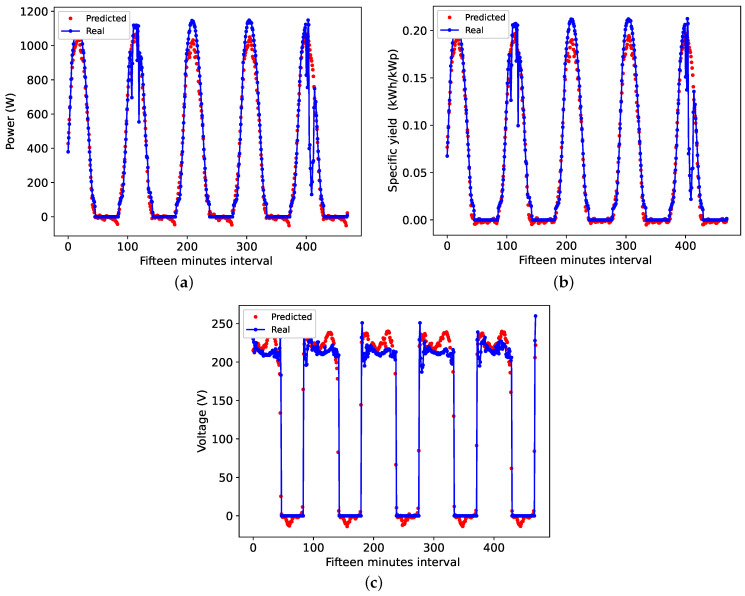
Forecast curve in 24 h: (**a**) Power, (**b**) efficiency and (**c**) Voltage.

Figure 11a–c depicts the individual outcomes of the 24 h horizon variable forecast, presenting a comparison between the TCN-predicted data and the actual data. Challenging aspects of accurately forecasting intermittent photovoltaic generation are commonly recognized. Notably, the second and fifth forecast days demonstrate substantial variations due to cloud presence, which are not observed in the forecast. The TCN’s behavior appears smooth, unlike the 15 min forecast, indicating the inability to predict cloud presence with a delay.

**Figure 12:**
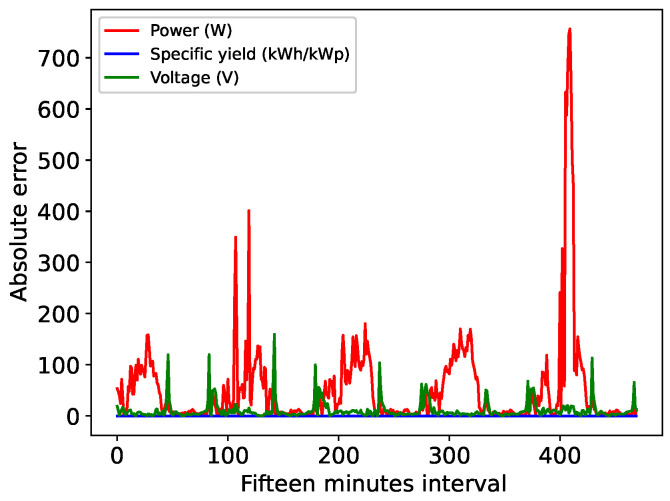
Absolute error for the 24 h TCN forecast.

Figure 12 presents the absolute error observed in the TCN’s 24 h horizon forecasts for power, efficiency and voltage. Substantial fluctuations in the estimated variables resulting from cloud cover on the second and fifth forecast days add complexity to the forecast process. Notably, the absolute error approached 800 W on the fifth day.

### 4.3. Sensitivity Analyses for TCN Forecast

To evaluate how the forecast horizon and the size of the dataset influence the results in order to evaluate under which circumstances the TCN method is robust and can be used. In this context, Table 9 displays the sensitivity analysis of TCN concerning variations in the dataset length for a 24 h delay.

Upon observing the values in Table 9, it is evident that as the dataset size increases, the MSE decreases while $R^{2}$ increases. Even with half the dataset (6 months), it is possible to achieve a good outcome with an MSE value of 0.0080 and an $R^{2}$ of 0.90. Another important analysis is how the forecast horizon influences the results. So, Table 10 presents the TCN sensitivity analysis regarding variations in the forecast horizon.

It is noticeable in Table 10 that as the forecast horizon increases, the MSE also increases while $R^{2}$ decreases. Specifically, for a 12 h forecast horizon, the MSE value is 0.0051 and $R^{2}$ is 0.95. Therefore, it should be noted that the methodology proposed in this paper was tested in real PV application, presents higher accuracy in different short-term time horizons, does not require long measurement periods (it presented satisfactory results with 6 months of data), with the only necessity being the radiance data as input and it can probably be used in any region and type of photovoltaic system.

### 4.4. Discussion

The limitations of the study include not using a meta-heuristic-based optimizer to define the hyperparameters, resulting in an empirical process within this methodology. Furthermore, the inclusion of temperature as an input in the networks could potentially enhance the results, provided that this temperature adequately represents the ambient temperature.

The sensitivity analysis allowed for verifying the robustness of the TCN concerning the forecast window, dataset size and, indirectly, the environmental variation caused by seasonal changes in irradiation. Through this analysis, it was observed that with more data, the forecast tends to improve. However, in this study, the dataset size and other meteorological variables acted as limiting factors.

## 5. Conclusions and Future Works

The performance of LSTM, BILSTM and TCN have been evaluated in this research paper with the aim to forecast the PV generation of an amorphous PV plant installed in the Technical Support Centre in the University Rey Juan Carlos (Madrid, Spain). For this, the PV current, voltage and efficiency are predicted using 15 min and 24 h forecast timesteps based on experimental historical data that correspond to one year. The results’ comparison shows that TCN presents better results than the other two techniques for 15 min and 24 h forecast in term of MSE and test execution time. Moreover, BiLSTM shows better results than LSTM in training and validation MSE.

The future work will integrate a self-attention mechanism into the TCN to enhance its performance, as it allows the model to selectively and proportionally focus on different parts of the input data. This addition will leverage the forecast obtained for optimizing energy management in autonomous PV plants for the upcoming day. Furthermore, new environmental variables will be introduced to enhance predictive accuracy and explore abrupt variations in these environmental conditions within the proposed methodology. Finally, another perspective of future works is the inclusion of an optimizer to define the ANN hyperparameters.

## Figures and Tables

**Table 1:** Parameter of the amorphous photovoltaic module.

Parameter	Module
Rated Power (wp)	60.0 Wp
Power Tolerance (%)	−5 to 10
Module Efficiency at STC (%)	6.34
Nominal voltage (V)	67.0
Nominal current (A)	0.9
Open-circuit voltage (V)	92.0
Short-circuit current (A)	1.19
Physical dimensions (mm)	960 × 990
Voltage/temperature coefficient (%)	0.0748
Current/temperature coefficient (%)	0.0752
Power/temperature coefficient (%)	−0.140

**Table 2:** Statistical analysis of dataset variables.

	Power	Spec. Yield	Voltage	Irradiance	Temperature
mean	243.44	0.04	107.47	199.65	19.59
std	358.57	0.07	110.03	297.69	14.38
min	0.00	0.00	0.00	0.00	−5.00
max	1329.12	0.25	324.00	1173.49	65.00

**Table 3:** Architecture chosen for the LSTM network on the 15 min and 24 h forecasting.

Layer	Type	Values
#1	Input	Dimension = 96 × 1
#2	LSTM	cells = 96, Activation = sigmoid
#3	LSTM	cells = 12, Activation = sigmoid
#4	Dense	Neurons = 24, Activation = sigmoid
#5	Dense	Neurons = 48, Activation = sigmoid, Dropout = 0.1
#6	Output	Neurons = 3, Activation = linear

**Table 4:** Architecture chosen for the BiLSTM network on the 15 min and 24 h forecasting.

Layer	Type	Values
#1	Input	Dimension = 96 × 1
#2	BiLSTM	cells = 96, Activation = sigmoid
#3	Dense	Neurons = 12, Activation = sigmoid
#4	Dense	Neurons = 24, Activation = sigmoid, Dropout = 0.05
#5	Output	Neurons = 3, Activation = linear

**Table 5:** Architecture chosen for the TCN network on the 15 min and 24 h forecasting.

Layer	Type	Values
Input	–	Dimension = 96 × 1
#1	TCN	Filters = 32, KernelSize = 6, Activation = ReLu, Dilations = [1, 2, 4, 8, 16, 32], NbStacks = 1
Output	Dense	Neurons = 3, Activation = linear

**Table 6:** MSE, $R^{2}$ and execution times of different models in 15 min forecasts.

Algorithm	LSTM	BiLSTM	TCN	*T* in *T* + 1
Training MSE (-)	0.0040	0.0038	0.0024	–
Validation MSE (-)	0.0051	0.0049	0.0039	–
Testing MSE (-)	0.0031	0.0032	0.0024	0.0053
Testing $R^{2}$ (-)	0.97	0.96	0.98	0.94
Test execution time (ms)	9	10	6	–

**Table 7:** MSE, $R^{2}$ and execution times of different models in 24 h forecasts.

Algorithm	LSTM	BiLSTM	TCN	*Day* in *Day* + 1
Training MSE (-)	0.011	0.010	0.0026	–
Validation MSE (-)	0.008	0.011	0.0075	–
Testing MSE (-)	0.007	0.010	0.0058	0.012
Testing $R^{2}$ (-)	0.93	0.90	0.95	0.84
Test execution time (ms)	10	11	7	–

**Table 8:** TCN MSE on the 15 min and 24 h delay dataset.

		Overall	Power	Spec. Yield	Voltage
TCN	15 min	0.0024	0.0020	0.0019	0.0032
TCN	24 h	0.0058	0.0055	0.0060	0.0061

**Table 9:** TCN sensitivity analysis considering dataset length variation on the 24 h delay.

Dataset Size	MSE	$R^{2}$
1/3	0.0106	0.79
1/2	0.0080	0.90
2/3	0.0062	0.93
1	0.0058	0.95

**Table 10:** TCN sensitivity analysis considering variations in the forecast horizon.

Forecast Horizon	MSE	$R^{2}$
15 min	0.0024	0.98
1 h	0.0034	0.97
12 h	0.0051	0.95
24 h	0.0058	0.95

## Data Availability

Data are contained within the article.
